# Choroidal Thickness in Acute Non-arteritic Anterior Ischemic Optic Neuropathy

**DOI:** 10.18502/jovr.v15i1.5946

**Published:** 2020-02-02

**Authors:** Homayoun Nikkhah, Mohadeseh Feizi, Naser Abedi, Saeed Karimi, Mehdi Yaseri, Hamed Esfandiari

**Affiliations:** ^1^Ophthalmic Research Center, Shahid Beheshti University of Medical Sciences, Tehran, Iran; ^2^Department of Ophthalmology, Torfeh Hospital, Shahid Beheshti University of Medical Sciences, Tehran, Iran; ^3^Department of Epidemiology and Biostatistics, School of Public Health, Tehran University of Medical Sciences, Tehran, Iran; ^4^Department of Ophthalmology, Northwestern University Feinberg School of Medicine, Chicago, USA

**Keywords:** Choroidal Thickness, Non-arteritic Ischemic Optic Neuropathy, Optic Nerve Head, Swept Source Optical Coherence Tomography

## Abstract

**Purpose:**

To compare the choroidal thickness in eyes with acute non-arteritic anterior ischemic optic neuropathy (NAION) with healthy contralateral eye and normal controls.

**Methods:**

Thirty-eight eyes with NAION, thirty-eight unaffected fellow eyes, and seventy-four eyes from 37 healthy, age- and sex-matched subjects were included in this prospective comparative case-control study. Choroidal thickness was measured by enhanced depth imaging (EDI) of spectral domain optical coherence tomography (SD-OCT). Peripapillary choroidal thickness (PCT) was measured at 1000 and 1500 μm from Bruch's membrane opening (BMO). Subfoveal choroidal thickness (SFCT) was measured in central subfoveal area, and 500 microns apart in temporal and nasal sides. Choroidal thickness among NAION eyes, uninvolved fellow eyes, and control eyes were compared.

**Results:**

The mean of PCT at 1000 μm was significantly thicker in NAION and fellow eyes compared to control eyes (169.7 ± 47, 154.4 ± 42.1, and 127.7 ± 49.9 μm, respectively, *P* < 0.001 and *P* = 0.42). The mean PCT at 1500 μm was also significantly thicker in NAION and fellow eyes compared to control eyes (178.6 ± 52.8, 162.6 ± 46.1, and 135.1 ± 59 μm, respectively, *P* = 0.007 and *P* = 0.048). The mean PCT at 1000 and 1500 μm was significantly greater in NAION compared to fellow eyes (*P* = 0.027 and *P* = 0.035, respectively). The mean of SFCT was significantly thicker in NAION compared to control eyes (*P* = 0.032); however, there was no significant difference between uninvolved fellow and control eyes (*P* = 0.248).

**Conclusion:**

Thicker choroidal thickness in acute NAION and uninvolved fellow eyes compared to normal eyes suggests a primary choroidal role in NAION pathophysiology.

##  INTRODUCTION

Although the role of choroid in several sight-threatening ocular conditions such as polypoidal choroidal vasculopathy or choroidal neovascularization^[1,2,3,4]^ is well-studied, it is only recently that the details of the choroid have been evaluated with enhanced depth imaging optical coherence tomography (EDI-OCT). EDI-OCT provides *in vivo* cross-sectional image of the choroidal layer.^[5]^ Since choroid is located adjacent to optic nerve and shares interconnected blood supply with optic disc, investigators have started to look at its possible role in two of the most common optic neuropathies, that is, non-arteritic anterior ischemic optic neuropathy (NAION) and glaucoma.^[6,7,8,9]^ NAION is the most common acute optic neuropathy^[10]^ and, as the name implies, it is presumed to be secondary to the vascular insufficiency within optic disc space, but the nature of the vasculopathy and its pathophysiologic mechanism is not definitely known yet.^[11]^

Evaluating choroidal thickness in acute NAION may help identify choroidal role in NAION and clarify if the choroidal thickness changes in NAION is a predisposing factor for this condition or the consequence of acute optic neuropathy.

The purpose of this case-control study was to evaluate the peripapillary choroidal thickness (PCT) and subfoveal choroidal thickness (SFCT) in eyes with acute NAION and compare them with uninvolved fellow eyes and eyes of healthy controls.

##  METHODS

This prospective comparative case-control study was carried out at Torfeh Hospital as a university-based tertiary eye center. The study protocol was approved by the Ethics Committee and Review Board of Shahid Beheshti University of Medical Sciences. All investigations were carried out in accordance with the Declaration of Helsinki. Patients with acute NAION and less than 14 days of clinical presentation were included from January 2014 to June 2015.

The diagnosis of NAION was based on the presence of a history of sudden painless visual loss associated with a relative afferent pupillary defect that was not attributed to any other ocular, neurologic, or systemic disease, optic disc swelling detected by biomicroscopic examination at the time of presentation and confirmed by peripapillary retinal nerve fiber layer thickness measurement using OCT, visual field testing compatible with ischemic optic neuropathy, and resolution of disc swelling and appearance of optic atrophy within two months.

The exclusion criteria were clinical symptoms of giant cell arteritis, high erythrocyte sedimentation rate (ESR) or C-reactive protein (CRP) level, any history or clinical evidence of retinal or neurologic disease, previous retinal procedure (e.g., laser photocoagulation), intraocular pressure (IOP) > 21 mmHg, or usage of glaucoma medication, presence of glaucomatous optic nerve damage, any other optic nerve disease, and bilateral NAION cases. Healthy volunteers from the optometry clinic with a refractive error in the range of –4 to +4, and no history of intraocular surgery were recruited as the control group. For all cases and controls, comprehensive ophthalmic examination including best corrected visual acuity (BCVA) assessment, slit-lamp examination, Goldmann applanation tonometry, gonioscopy, fundus examination, and perimetry (Humphrey visual field analyzer; model 750; Carl Zeiss Meditec, Dublin, California, USA) were performed. Axial length was measured using IOL Master (ZEISS IOLMaster, Carl Zeiss Meditec AG, Germany).

##  OCT imaging

All OCT measurements were performed using Heidelberg spectral domain OCT (Spectralis; Heidelberg Engineering, Heidelberg, Germany) by the same experienced operator. For each eye, three OCT images were acquired.

**Figure 1:**
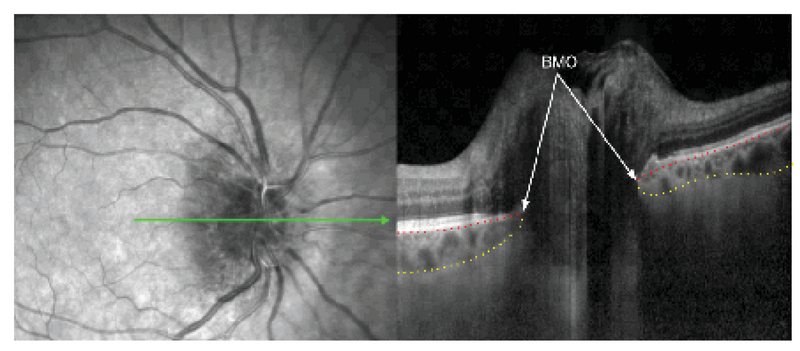
Delineated SD-OCT from affected eye: Bruch's membrane (dotted red line), anterior sclera (dotted yellow line), BMO, Bruch's membrane opening.

PCT was measured using horizontal and vertical raster EDI-OCT scans centered at optic nerve head (ONH) [Figure 1]. SFCT was determined using horizontal raster EDI-OCT scans centered at the fovea. Macular OCT scan: 25 horizontal optical coherent tomographic sections were obtained in a 20${}^{\mu }\times$ 20${}^{\mu }$ rectangle centered around the fovea. Three circular lines demonstrating 1, 3, and 6 mm of the central macula (Early Treatment Diabetic Retinopathy Study (ETDRS) macular grid) were obtained. The data of central 1 mm circle was defined as central macular thickness (CMT). In addition, ganglion cell-inner plexiform layer (Gc-IPL) thickness in central 1 mm circle and inner macular area (between the central and middle circles) were measured in four superior, inferior, temporal, and nasal quadrants, using instrument segmentation software. It has been shown that the Spectralis software has an excellent reproducibility in the measurement of each individual retinal layer.^[12]^

**Figure 2:**
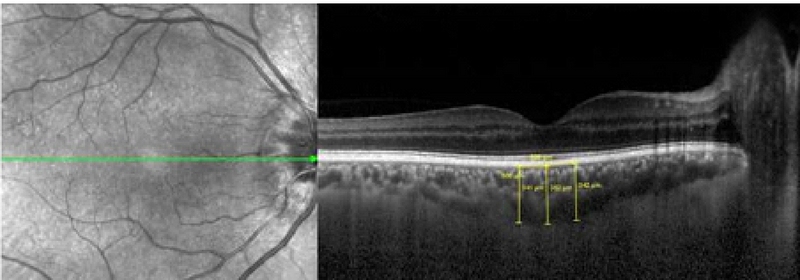
Choroidal thickness at the center of the fovea and 500 μm from the center at temporal and nasal side.

Choroidal thickness was measured along the perpendicular distance from posterior border of retinal pigment epithelium to anterior scleral border (choroidal–scleral interface) using the OCT manual measurement tool. For peripapillary choroid, we measured the choroidal thickness at 1000 (±10) and 1500 (±10) μm from Bruch's membrane opening (BMO) in superior, inferior, temporal, and nasal parts on vertical and horizontal raster scans (eight points). To compare the choroidal thickness at 1000 and 1500 μm from BMO among the eyes with NAION, uninvolved fellow eyes and control eyes, we calculated the mean of measurements at 1000 and 1500 μm in superior, inferior, temporal, and nasal parts. In the subfoveal area, choroidal thickness was measured at the foveal center as well as 500 (±5) μm from the foveal center in both temporal and nasal sides [Figure 2]. The mean of these three measurements was defined as the mean SFCT. To better determine the choroidal–scleral interface, we changed the contrast and resolution scales of the OCT device several times until we could find the exact borders. Considering reports of diurnal variation in choroidal thickness, all OCT images were carried out between 10 and 12 am. All choroidal measurements were done by the same surgeon (HN) and re-evaluated by two other co-authors (MF, HE).

##  Statistical method

To present data, we used mean, standard deviation, median, and range. To assess normal distribution of quantitative data, we used Kolmogorov–Smirnov test and Q–Q plot. To compare the results between groups at baseline, we used *t*-test, Mann–Whitney test, and Chi-square test. Considering the probability correlation of the measures, Generalized Estimating Equation (GEE) analysis was used to compare the groups. Furthermore, adjusted results for hypertension and diabetes were obtained using another GEE model. To consider the multiple comparisons, issue in pairwise comparisons, we used the Bonferroni method. The correlation of variables was obtained by the Pearson method. All statistical analyses were performed by SPSS software (IBM Corp. Released 2014. IBM SPSS Statistics for Windows, Version 23.0. Armonk, NY: IBM Corp.). All tests were two-sided and *P* < 0.05 was considered statistically significant.

##  RESULTS

Among 46 enrolled eyes, 38 eyes with acute NAION were included for final analysis. Eight eyes were excluded because of poor-quality OCT and inability to determine choroidal–scleral junction. Thirty-eight uninvolved fellow eyes and seventy-four eyes of 37 healthy, age- and sex-matched subjects were included as the control group. The mean age of NAION patients and control subjects were not significantly different (62 ± 11 and 61 ± 30 years, respectively, *P* = 0.95). The mean interval between the initiation of symptoms and OCT acquisition was 6.7 ± 3.7 days (range, 1 to 14). There was no difference between NAION, uninvolved contralateral, and control eyes regarding spherical equivalent (*P* = 0.64) and mean axial length (*P* = 0.87). Affected cases were more likely to have diabetes mellitus (DM), but the prevalence of systemic hypertension was comparable in both affected and healthy individuals [Table 1].

**Table 1:** Demographic data of NAION patients and healthy control subjects


		**Study groups**			**P**
		**NAION eyes**	**Uninvolved fellow eyes**	**Control eyes**	
Number (n)		38	38	74 (37)	
Age (Years)	Mean ± SD	62 ± 11	61 ± 30	0.959†
	Median (range)	61 (30 to 87)	87 (62 to 10)	
Sex	M	20 (52.6%)	18 (48.6%)	0.525*
	F	18 (47.4%)	19 (51.4%)	
Interval (Days)${}^{1}$	Mean ± SD	6.74 ± 3.82		
	Median (range)	6.5 (1 to 14)		
DM	No	21 (55.3%)	29 (78.4%)	0.010*
	Yes	17 (44.7%)	8 (21.6%)	
HTN	No	21 (55.3%)	27 (73.0%)	0.077*
	Yes	17 (44.7%)	10 (27.0%)	
SE	Mean ± SD	0.39 ± 1.11	0.46 ± 1.16	0.27 ± 0.99	0.641†
	Median (range)	0.38 (–2 to 3.75)	0.5 (–2 to 3.5)	0.25 (–3 to 3)	
Axial length	Mean ± SD	22.74 ± 0.76	22.74 ± 0.78	22.83 ± 0.84	0.873†
	Median (range)	22.98 )	22.88 (21.05 to 23.85)	22.97 (21.09 to 24.31)	
1: Interval between initial symptom and ophthalmic evaluation					
DM, diabetes mellitus; HTN, systemic hypertension; NAION, nonarteritic anterior ischemic optic neuropathy; SE, spherical equivalent					
†Based on *t*-test					
‡Based on Mann–Whitney test					
Based on Chi-square test					

**Table 2:** Intraclass correlation coefficients for PCT and SFCT in a random subset of 15 eyes


	**Intraobserver Variability (95% Confidence Interval)**	**Interobserver Variability (95% Confidence Interval)**
PCT 1000 (temporal)	0.934 (0.841–0.973)	0.874 (0.710–0.948)
PCT 1000 (nasal)	0.971 (0.940–0.992)	0.963 (0.920–0.984)
PCT 1500 (temporal)	0.966 (0.914–0.986)	0.933 (0.831–0.973)
PCT 1500 (nasal)	0.968 (0.922–0.985)	0.941 (0.853–0.974)
SFCT (temporal)	0.959 (0.910–0.981)	0.981 (0.960–0.992)
SFCT (nasal)	0.992 (0.951–0.996)	0.961 (0.912–0.983)
PCT, peri-papillary choroidal thickness; SFCT, subfoveal choroidal thickness									

**Table 3:** Measurements of PCT in different areas in NAION, uninvolved and control eyes


	**NAION eyes(1)**	**Uninvolved eyes(2)**	**Control eyes(3)**	**Diff (1 vs 2)**	P1*	**Diff (1 vs 3)**	P2*	**Diff (2 vs 3)**	P3*
	**Mean ± SD**	**Mean ± SD**	**Mean ± SD**			
PCT S 1000	173.2 ± 59.4	164.9 ± 47.4	135.7 ± 57.3	8.2	1.000	37.4	0.007	29.2	0.065
PCT I 1000	151.2 ± 62.4	131.5 ± 47.5	112.2 ± 51.7	19.7	0.076	39.0	0.012	19.3	0.112
PCT T 1000	181.5 ± 69.9	167.9 ± 53.9	131.8 ± 58.7	13.7	0.429	49.7	0.001	36.1	0.043
PCT N 1000	172.8 ± 61.9	153.5 ± 52.2	131.2 ± 54.9	19.3	0.027	41.6	0.005	22.3	0.221
Mean PCT 1000	169.7 ± 47	154.4 ± 42.1	127.7 ± 49.9	15.2	0.027	41.9	< 0.001	26.7	0.042
PCT S 1500	175.3 ± 69.6	165.5 ± 51.3	141.2 ± 69.4	9.8	1.000	34.0	0.054	24.3	0.287
PCT I 1500	155.5 ± 71.4	132.7 ± 47.4	113.8 ± 62.5	22.8	0.059	41.7	0.015	18.9	0.395
PCT T 1500	208.7 ± 80.6	190.5 ± 67.7	144.9 ± 64.7	18.3	0.301	63.9	< 0.001	45.6	0.012
PCT N 1500	172.7 ± 64.1	161.7 ± 55.6	140.5 ± 64	11.0	0.314	32.2	0.073	21.2	0.442
Mean PCT 1500	178.6 ± 52.8	162.6 ± 46.1	135.1 ± 59	16.0	0.035	43.5	0.007	27.5	0.048
PCT, peri-papillary choroidal thickness; NAION, non-arteritic anterior ischemic optic neuropathy; S, superior; I, inferior; T, temporal; N, nasal; 1000, at 1000micron from Bruch's membrane opening; 1500, at 1500micron from Bruch's membrane opening; SD, standard deviation; Diff, difference									
P-for pairwise comparison, based on post hoc analysis of GEE, adjusted for the multiple comparison by Bonferroni method									

**Table 4:** Measurements of GC-IPL, RFNL, SFCT and CMT in different areas in study groups (NAION, uninvolved, and control eyes)


	**NAION eyes**	**Uninvolved eyes**	**Control eyes**	**Diff (NAION vs Uninvolved eyes)**	P1*	**Diff (NAION vs Control eyes)**	P2*	**Diff (Uninvolved vs Control eyes)**	P3*
	**Mean ± SD**	**Mean ± SD**	**Mean ± SD**			
GC-IPL C	20.1 ± 14.7	13.2 ± 5.3	13.1 ± 3.4	6.8	< 0.001	6.9	< 0.001	0.1	1.000
GC-IPL S	46.2 ± 14.1	45.8 ± 13	51.9 ± 5.4	0.4	1.000	–5.6	0.045	–6.0	0.019
GC-IPL I	45.8 ± 12.1	46.2 ± 12.8	51.4 ± 5.6	–0.3	1.000	–5.5	0.018	–5.2	0.033
GC-IPL T	41 ± 12.7	43.9 ± 13	45.7 ± 5.8	–2.9	0.687	–4.7	0.078	–1.8	1.000
GC-IPL N	45.7 ± 13.8	45.8 ± 12.5	49.6 ± 5.3	–0.1	1.000	–3.9	0.173	–3.8	0.167
SFCT C	286.7 ± 72.7	271 ± 68.2	237.2 ± 89.1	15.7	0.352	49.4	0.034	33.7	0.234
SFCT T 500	267.3 ± 72.9	250.8 ± 71	219.8 ± 99.6	16.6	0.701	47.5	0.032	30.9	0.323
SFCT N 500	271 ± 76.3	253.1 ± 63.2	220.6 ± 94.1	17.8	0.345	50.3	0.035	32.5	0.245
Mean SFCT	275 ± 71.7	258.3 ± 65	225.9 ± 91.2	16.7	0.123	49.1	0.038	32.4	0.248
CMT	339.9 ± 131	274.8 ± 54.1	266 ± 25	65.1	< 0.001	73.9	< 0.001	8.8	1.000
GC-IPL, ganglion cell-inner plexiform layer; SFCT, subfoveal choroidal thickness; CMT, central macular thickness; NAION, non-arteritic anterior ischemic optic neuropathy; C, central; S, superior; I, inferior; T, temporal; N, nasal; T 500, 500micron temporal to fovea; N 500, 500micron nasal to fovea; SD, standard deviation; Diff, difference.									
P-for pairwise comparison, based on post hoc analysis of GEE, adjusted for the multiple comparison by Bonferroni method									

**Table 5:** PCT, GC-IPL, and SFCT values adjusted for hypertension and diabetes mellitus


	**Diff (NAION vs Control eyes)**	**SE**	P*	**Diff (Uninvolved vs Control eyes)**	**SE**	P*
PCT S 1000	37.966*	11.5	0.007	31.075*	11.5	0.032
PCT I 1000	44.470*	11.0	< 0.001	23.5	11.0	0.121
PCT T 1000	50.309*	12.6	< 0.001	35.958*	12.6	0.019
PCT N 1000	42.085*	11.6	0.001	22.6	11.6	0.167
**Mean**	43.708*	9.7	< 0.001	28.282*	9.7	0.025
PCT S 1500	38.097*	13.5	0.013	28.3	13.5	0.127
PCT I 1500	45.619*	12.8	0.012	22.9	12.8	0.237
PCT T 1500	63.130*	14.6	< 0.001	44.698*	14.6	0.013
PCT N 1500	33.351*	12.9	0.044	23.4	12.9	0.321
**Mean**	45.049*	11.3	< 0.001	29.826*	11.3	0.034
GC-IPL C	6.924*	1.7	< 0.001	0.0	1.7	1.000
GC-IPL S	–5.411*	2.2	0.043	–5.708*	2.2	0.034
GC-IPL I	–6.383*	2.0	0.005	–5.437*	2.0	0.025
GC-IPL T	–5.0	2.1	0.057	–1.9	2.1	1.000
GC-IPL N	–3.3	2.1	0.331	–2.9	2.1	0.612
Mean GC-IPL	–2.7	1.7	0.367	–3.2	1.7	0.201
SFCT C	49.586*	18.2	0.025	33.9	18.2	0.201
SFCT T 500	50.091*	17.4	0.012	34.4	17.4	0.172
SFCT N 500	75.364*	15.5	< 0.001	8.8	15.5	1.000
Mean SFCT	49.838*	16.8	0.012	34.9	16.8	0.123
PCT, peripapillary choroidal thickness; GC-IPL, ganglion cell- inner plexiform layer; SFCT, Subfoveal choroidal thickness; Diff, difference; SE, standard error; NAION, non-arteritic anterior ischemic optic neuropathy; C, central; S, superior; I, Inferior; T, temporal; N, nasal; T 500, 500 μm temporal to fovea; N 500, 500 μm nasal to fovea; SE, standard error; Diff, difference						
P-for pairwise comparison adjusted for the HTN and diabetes, based on post hoc analysis of GEE, multiple comparison considered by Bonferroni method						

Intraobserver and interobserver reproducibility of PCT and SFCT are shown in Table 2. There was excellent reproducibility in the intraobserver and interobserver tests (ranging from 0.934 to 0.992 for the intraobserver and from 0.874 to 0.961 for the interobserver reproducibility).

The mean PCT at 1000 μm from BMO (PCT1000) was 169.7 ± 47, 154.4 ± 42.1, and 127.7 ± 49.9 μm in NAION eyes, uninvolved fellow eyes, and control eyes, respectively. PCT1000 was significantly thicker in the involved eyes and uninvolved fellow eyes compared to the control eyes (*P* < 0.001 and *P* = 0.042, respectively). Moreover, PCT1000 was significantly thicker in involved eyes than uninvolved fellow eyes (*P* = 0.025) [Table 3].

The mean PCT at 1500 μm from BMO (PCT1500) was also significantly thicker in the NAION and uninvolved fellow eyes than the control eyes (*P* = 0.007 and *P* = 0.048, respectively). In addition, PCT1500 was significantly thicker in the eyes with NAION than the uninvolved fellow eyes (*P* = 0.035) [Table 3].

The mean SFCT was 275 ± 71.7, 258.3 ± 65, and 225.9 ± 91.2 μm in eyes with NAION, uninvolved fellow eyes, and control eyes, respectively. The mean SFCT was thicker in the eyes with NAION than the control eyes (*P* = 0.038), however, there was no statistically significant difference between the fellow and control eyes (*P* = 0.248) [Table 4].

CMT was significantly higher in the NAION than the uninvolved and control eyes (*P* < 0.001 and *P* < 0.001, respectively), but there was no significant difference between the fellow and control eyes (*P* = 1.000).

Central Gc-IPL thickness was significantly thicker in the eyes with NAION eyes than the uninvolved fellow and control eyes (*P* < 0.001 for both). Central Gc-IPL thickness in the eyes with NAION was negatively correlated with the BCVA (r = 0.31, *P* = 0.006). There was no statistically significant difference among the NAION, uninvolved fellow, and control eyes regarding the Gc-IPL thickness in the inner macular area [Table 4].

The differences persisted once adjusted for DM and systemic hypertension [Table 5].

##  DISCUSSION

In the present study, we found significantly thicker choroid in both acute NAION and unaffected fellow eyes compared to controls, both in peripapillary and subfoveal areas.

All previous studies evaluated the PCT in the chronic phase of NAION.^[8,9]^ This study is the first one that measured PCT in acute phase. By adjusting the contrast and resolution scale of the OCT device, we were able to measure choroidal thickness despite edema and disc swelling.

Although the exact mechanism of NAION is yet to be understood, ischemia and the so called “compartment syndrome” in optic disc space are the main determinants for the loss of visual function.^[14,15]^

Whatever the mechanism of vascular insufficiency in NAION, persistent hypoperfusion is required for the development of ischemic optic neuropathy. The paraoptic branches of the short posterior ciliary arteries are responsible for ONH blood supply and the infarction in NAION is suggested to be predominantly located in the retrolaminar portion of the ONH.^[16]^ Therefore, since the choroid mainly supplies – to a limited extent – more anterior optic nerve head regions, it is less likely that choroid has a substantial role in the pathogenesis of NAION. However, several studies reported changes in choroidal thickness in NAION,^[7][8,9]^ which is consistent with the findings of the current study.

Fard et al evaluated the PCT in chronic NAION and reported increased thickness in both affected and unaffected fellow eyes compared to normal controls.^[8]^ Our finding also matches the finding of Nagia et al that observed increased thickness of the PCT, especially 1000 to 1500 μm from BMO, in chronically affected NAION and contralateral eyes compared to controls.^[10]^ Surprisingly, the mean PCT in the affected eyes in our study was lower than the aforementioned studies with chronic NAION. Although one should be cautious to draw any conclusion from these cross-sectional studies, chronic cases were expected to have thinner choroid compared to the eyes at the acute stage if choroidal thickening was secondary to ischemic damage. However, longitudinal studies are needed to evaluate the changes of choroidal thickness following the ischemic damage.

The lack of consistent choroidal filling defect in angiographic studies^[17]^ further reinforces the primary role of choroid in the development of NAION.

Crowded disc is known as a predisposing factor for NAION, although the mechanism by which it contributes to ischemia is unclear.^[11]^ Increased PCT may be a component of crowded disc morphology as suggested by Fard et al.^[8]^ They hypothesized that the thicker choroid may strain the optic nerve axon and its vessel within the narrow central cup and further restrict the crowded optic nerve space.

Thicker peripapillary choroid in NAION eyes may be a structurally predisposing factor or may be a compensatory mechanism due to ischemia. Thicker choroid, both in acute NAION and uninvolved fellow eyes, rationally suggests the role of choroid as a predisposing factor rather than a compensatory mechanism. Moreover, if increased choroidal thickness was a compensatory factor, it must be decreased after the acute phase, but as mentioned earlier, the result of previous studies in the chronic phase also showed thicker choroid.^[8,9]^ The NAION progresses through asymptomatic “incipient NAION” to full blown ischemic neuropathy with characteristic clinical presentation in more than two-third of cases^[18,19]^. By further contributing to “compartment syndrome” in already constricted scleral canal, increased choroidal thickness may have a role in the development of symptomatic axonal damage. In that sense, increased choroidal thickness acts as a “catalyzer” for the progression of ischemic optic neuropathy.

Moreover, Beijing Eye Study showed no association between SFCT and optic disc size in normal subjects, therefore choroidal thickness could affect the incidence and progression of NAION independent of optic disc size.^[20]^ Thicker choroid may also be a sign of local auto-regulation dysfunction of the choroidal vessel.^[21]^

In contrast to our study and others,^[8,9]^ Garcia-Basterra et al^[10]^ reported thinner PCT in NAION and unaffected fellow eyes compared to normal subjects. The differing results may be because they did not match the refractive state of control subjects and NAION patients, and also they evaluated PCT in chronic phase of AION.

Gonal et al^[22]^ reported no difference between sub-macular choroidal thickness in acute and chronic NAION eyes and control subjects, they included subjects with optic disc edema that had occurred in a time period of less than one month as an acute NAION, but we included patients with initial symptoms occurring less than 14 days; they also measured choroidal thickness in farther points from the foveal center. Interestingly, Schuster et al reported thinner SFCT in the affected and non-affected fellow eyes of patients with NAION.^[7]^ Although the authors considered the enrolled cases as acute NAION, the duration of the disease that was considered acute is not reported in their paper, so we cannot confidently compare our findings with their results. Besides, they used one horizontal line passing through fovea and CT was measured on 1 point on this line. We argue that measuring choroidal thickness at 1 selected point, which is performed manually, could be affected by irregularities of choroidal-scleral boundary.^[23]^ In addition, not all factors affecting the choroidal thickness measurement were adjusted in that study.

The thickening of central macular region in our study is in line with previous reports that have shown subretinal fluid accumulation in recent onset NAION.^[24,25,26]^ Since macular OCT is not routinely obtained in NAION, the exact incidence of macular edema is unknown in these eyes but it is estimated to range from 10 to 50%.^[23]^ There is no longitudinal study that has evaluated the course of macular edema after the NAION and its effect on the final visual acuity.

In the present study, we found that the Gc-IP layer in the central 1 mm was significantly thicker in NAION eyes compared to uninvolved fellow eyes and control eyes, while in all quadrants of 1 to 3 mm annulus, there was no significant difference among NAION, uninvolved fellow, and control eyes in terms of Gc-IP layer thickness. The thickening of Gc-IPL in the central 1 mm may be due to intracellular edema due to greater ischemic stress on central fovea in the early phase of NAION and contributes to vision deterioration. Fard et al^[27]^ reported that parafoveal Gc-IPL thinning present in chronic NAION eyes was associated with worse vision compared to glaucoma patients in which central vision is preserved and has no Gc-IPL thinning in this area. Parks et al reported the initial thinning of Gc-IP layer in the macula occurring 46.1 ± 23.2 days after the onset of NAION symptoms.^[28]^ We measured Gc-IPL thickness in the period of less than two weeks from initial symptoms, which showed thickening only in the affected eyes. The ischemia induced axoplasmic flow stasis results in axonal swelling^[28]^ and is considered an early event in the “sequence of events” in NAION.^[29]^ In fact, this early axonal swelling is the stage where timely interventions such as anti-inflammatory and neuroprotective agents proved to be promising.^[14,31,32]^

This study has several limitations. The sample size was relatively small but is comparable to previous studies on this subject and necessitates further longitudinal studies to clarify whether choroidal thickening precedes NAION or it occurs following the ischemic incidence.

Although anterior scleral border was delineated manually, intra- and interobserver reproducibility were excellent, which is in line with the high reproducibility reported in previous studies.^[8,9]^ Furthermore, recent endeavors to quantify choroidal thickness with automated segmentation have shown excellent consistency with manual labeling.^[33]^ Choroidal thickness has not been adjusted for IOP in our study and it may have caused some random errors, although the induced bias may be far from significance.

In summary, we showed that the boundaries of the choroid in acute NAION could be delineated by excellent intra- and interobserver reproducibility. Moreover, our finding of increased choroidal thickness in both eyes of the affected patients compared to controls lends support to the theory of primary choroidal role in the development of NAION.

##  Financial Support and Sponsorship

Nil.

##  Conflicts of Interest

There are no conflicts of interest.
